# Clinical characteristics and health care received among patients with type 2 diabetes attending secondary and tertiary healthcare facilities in Mwanza Region, Tanzania: a cross-sectional study

**DOI:** 10.1186/s12913-020-05407-y

**Published:** 2020-06-10

**Authors:** Mariam J. Munyogwa, Reuben William, Stephen M. Kibusi, Nyasiro S. Gibore

**Affiliations:** 1 Department of Public Health, University of Dodoma, P. O. Box 395, Dodoma, Tanzania; 2 Department of Research, Sumve School of Nursing, P. O. O. Box 7, Mantare, Mwanza, Tanzania

**Keywords:** T2DM, Healthcare facility, Obesity, Hyperglycemia, Hypertension, Diabetes-related complications, Consultation, Nurse, Dietitian, Clinician

## Abstract

**Background:**

Tanzania is among the sub-Saharan African countries facing a tremendous increase in the burden of type 2 diabetes mellitus. In order to provide diabetes health care services, the government has established diabetes care clinics in secondary and tertiary healthcare facilities. However, previous studies have demonstrated a disparity in availability of supplies and equipment for provision of diabetes health care services at these healthcare facilities. This study aims to assess the clinical characteristics and health care received among patients with type 2 diabetes attending secondary and tertiary healthcare facilities in Mwanza Region, Tanzania.

**Methods:**

A cross-sectional study was conducted in Mwanza Region from June to September, 2018.Three hundred and thirty patients were selected by systematic random sampling from three healthcare facilities. A structured questionnaire was utilized to collect information on patient characteristics, health care received and patient perception of care. Patient blood pressure, blood glucose, weight and height were measured during the study. Percentages, chi-square tests and multivariable analysis were conducted to obtain the proportions, make comparisons and determining the correlates of tertiary-level healthcare facility.

**Results:**

Approximately half of respondents (54.5%) were from secondary healthcare facilities. The prevalence of hypertension (63.3%), hyperglycemia (95.8%) and obesity (93.3%) were high. The prevalence of hyperglycemia was slightly higher at secondary-level healthcare facility (*p* = 0.005). The proportion of respondents recently diagnosed with diabetes (≤ 10 years) was significantly higher at tertiary-level healthcare facility (*p* = 0.000). The prevalence of diabetes related complications was higher at tertiary-level healthcare facility (80.7% versus 53.3%, *p* = 0.000). Assessments of body weight, blood pressure, blood glucose, feet and eye examination were conducted on a monthly basis at all facilities. None of the respondents had undergone lipid profile testing. All of the respondents (100%) received care from a nurse during diabetes clinic visits and half of the respondents (49.7%) also received care from a clinician. Relatively young patients, married and recently diagnosed patients were more likely to attend clinic at tertiary facilities. Tertiary-level healthcare facilities were more likely to have patients with complications and to have a dietitian available at the clinic.

## Background

Worldwide, prevalence of diabetes mellitus (DM) is increasing and the rate of increase is higher in developing than developed countries [1]. According to the 2013 International Diabetes Federation (IDF) report, 20 million people are estimated to have DM in sub-Saharan Africa (SSA) and the number is expected to double by 2035 if appropriate preventive measures are not undertaken [2]. Tanzania is among five SSA countries with a large number of people living with DM [3]. It is estimated that approximately 822,800 people in Tanzania are living with DM and most are found in urban areas [4–6]. Other SSA countries with large numbers of cases of diabetes include: South Africa (2,286,000), Democratic Republic of Congo (DRC) (1,762,900), Nigeria (1,564,700) and Ethiopia (1,333,200) [3].

People with diabetes can live long and healthy lives if the disease is detected early and well managed. Good management using a standardized protocol can potentially prevent complications and premature death resulting from diabetes and co-morbidities [1]. However, in most SSA countries [1] and Tanzania in particular [7–10], the availability of basic technology for diagnosis and management of diabetes remains a major challenge. This contributes to the higher prevalence of complications [1, 11–14] and high mortality [1, 15] among people with diabetes in the region. SSA countries have limited resources and face double burden of disease; whereby communicable diseases such as HIV/AIDS, tuberculosis and malaria are compounded by emerging non-communicable diseases such as diabetes [15]. Similar to other non-communicable diseases, diabetic care suffers a critical shortage in terms of human resources, materials and financing [7–10].

In Tanzania, diabetes treatment and health care services are currently available in clinics established in all hospitals that have varying levels of capacity to provide such services. Tanzanian healthcare facilities are divided into three levels: primary healthcare facilities are at the community level and include dispensaries and health centers; secondary healthcare facilities which include district and regional hospitals and tertiary healthcare facilities which include referral and specialized hospitals [7, 8]. Regarding provision of diabetes health care services, primary healthcare facilities lack capacity to formally diagnose diabetes or provide diabetes care or treatment [7, 8]. Diagnosis and management of diabetes is carried out at secondary and tertiary-levels healthcare facility where equipment and supplies for diabetes care services are available [7, 8, 10]. According to the healthcare facility referral lines, patients are first seen at primary-level healthcare facilities. Patients suspected of having diabetes are then referred to secondary healthcare facilities for diagnosis and confirmation. At secondary-level healthcare facility, confirmed diabetes cases are referred to specialty diabetes clinics where the patient is registered, initiated into treatment and monitored through specified scheduled clinics. Only patients without complications can be managed at secondary-level healthcare facility. Patients with complications are further referred to tertiary-level healthcare facility for advanced medical care [7]. However, for the purpose of attending routine DM clinics, the patient may decide to choose any healthcare facility based on accessibility. This approach seeks to reduce barriers such as long distances to the hospital, financial constrains [8] and long queues at the clinics [10]. It is thought that if care is restricted to one facility it might limit patient access to the healthcare facility [7, 8, 10] and contribute to poor health outcomes and premature death [16].

Previous studies in Tanzania have demonstrated differences in availability of diabetes related health care services including medical equipment, qualified staff, essential diabetes medicines and diabetes educational materials by different levels of healthcare facilities [7–10]. Such differences affect the quality of diabetes services provided by those healthcare facilities [7, 8, 10]. The health condition of a patient with diabetes can be influenced by the quality of health care received at the healthcare facility [1]. There is limited information about the health condition of patients with diabetes receiving health care services at different levels of healthcare facilities in Tanzania. Most studies on diabetes in Tanzania have focused on diabetes prevalence and risk factors [4–6, 17–19] and on diabetes-related complications [11–14]. Therefore, this study was designed to assess the clinical characteristics and health care received among patients with type 2 diabetes (T2DM) attending secondary and tertiary healthcare facilities in Mwanza Region, Tanzania.

## Methods

### Study area

This study was conducted in Mwanza Region, Tanzania. Administratively, the region is comprised of seven districts and has a total population of 2.77 million. The annual population growth rate is 3.0% [20]. The largest ethnic group is the Sukuma tribe that constitutes more than 90% of the total population. Other ethnic groups include: Zinza, Kerewe, Jita, Sumbwa, Haya, Luo and Nyamwezi tribes [21]. Swahili is the national language [21], and in this study all respondents were able to speak itclearly. Prevalence of diabetes mellitus ranges from 1.9 to 11.9% [6, 17]. Within the region, diabetes health care services are available at all district hospitals, one regional hospital (Sekou Toure Hospital) and one consultant and specialized hospital (Bugando Medical Centre (BMC)).

### Study design and study population

This study was a cross-sectional study. Study population was all type 2 diabetes patients attending DM clinics care at secondary and tertiary healthcare facilities in Mwanza Region. The study was conducted from June to September, 2018.The followings were excluded from this study: Patients below 18 years old, patients who have attended diabetes clinics for less than three times since diagnosis, pregnant or lactating women and very ill patients. Eligible patients for the study were determined by using patient medical report and the information obtained from the healthcare provider (HCP) at the clinic.

### Sample size and sampling procedures

Sample size was calculated by using the formula $n={z}^2p\frac{1-p}{e^2}$ whereby: n = sample size, z = 1.96, *p* = 71.2% [22] and e = 5%. Three out of nine healthcare facilities were selected for the study as follows: Two healthcare facilities, Bugando Medical Center (BMC) and Sekou Toure Regional Hospital were selected by purposive sampling technique and one healthcare facility (Ngudu District Hospital) was selected by simple random sampling. BMC and Sekou Toure Hospital are located in urban settings in the city of Mwanza. BMC is the tertiary healthcare facility for the Lake and Western zones of the United Republic of Tanzania. Diabetes care clinics are conducted twice per week and about 85 patients attend clinic each day. Sekou Toure is the Regional Hospital for Mwanza Region. Diabetes clinics are conducted twice per week and about 105 patients attend clinic per day. Ngudu District Hospital, in contrast, is located 90 km away from Mwanza City. The diabetes clinics are conducted on daily basis with an average of 9 patients attending the clinic per day.

Patients were selected by systematic random sampling technique. The selection was done based on the sitting arrangement at the waiting room. The first patient of the day was selected followed by every third patient until the end of the day. If the patient selected didn’t meet inclusion criteria, the next patient was selected without replacement. A total of 330 respondents were selected and agreed to participate in the study.

### Data collection

Data was collected using structured questionnaires. The questionnaire was developed by the researchers after an intensive literature review focusing on the objectives of this study (see Additional file 1). Afterward, the developed questionnaire was translated to Swahili language, pre-tested and modified accordingly to suit the current study. The questionnaire consisted of six pages and four sections namely: 1. Demographic characteristics, 2. Clinical characteristics, 3. Perceptions about diabetes health care received and 4. Health assessments performed during diabetes care clinic visit.

Demographic characteristics assessed included age of the patient, sex, marital status, type of residence, level of education and employment status. Clinical characteristics included were, blood pressure, random blood glucose, body weight and height, duration of the disease, presence of diabetes related disease and glucometer ownership. Measurements of blood pressure, random blood glucose, body weight and height were performed using a standard procedure.

Blood pressure (BP) was measured by using a sphygmomanometer and recorded in millimeters of mercury (mmHg). Measurement was taken while the patient was in sitting position and was performed twice at an interval of at least five (5) minutes. Hypertension was defined as SBP ≥ 140 and/or DBP of ≥90 mmHg or use of hypertensive medication [23].

Body weight was measured without shoes and with minimal clothing by using a SECCA® scale. It was recorded to the nearest 0.5 kg. Height was measured without shoes to the nearest 0.5 cm by a rigid stadiometer that was fitted together with the weighing scale. Body mass index (BMI) was calculated and categorized as non-obese (< 30 kg/m^2^) and obese (≥ 30 kg/m^2^). Random blood glucose was measured using a glucoplus machine. One drop of capillary blood was used to measure blood glucose concentration. Measurement was performed immediately after sample withdrawal. Hyperglycemia was defined as blood glucose concentration of ≥11.1 mmol/l [23]. The instruments used for measurements of blood pressure, random blood glucose, body weight and height were provided by the research team.

Duration of the disease, diabetes related diseases and glucometer ownership were assessed using patient’s medical history file and through face to face patient interview. We used the standard treatment guidelines and the National Essential Medicine List [23] to assess the health care services received during routine diabetes care clinic visit. For the purpose of this study the following health care services were assessed: measurements of body weight and height, blood glucose and lipid profile testing, blood pressure, foot and eye examination and provision of diabetes health education.

The research team was comprised of two trained researchers and one health care provider at Ngudu District Hospital and two health care providers at BMC and Sekou Toure Hospital. The health care providers also worked at the respective diabetes clinics. One day prior to data collection, the local health care provider was oriented and prepared for the exercise by a researcher. During data collection, the researcher was responsible for administering the questionnaire and measuring weight and height while the local health care provider was responsible for examination of blood pressure, blood glucose testing and providing feedback to the respondent based on the results obtained. Each measurement performed was recorded immediately into the questionnaire. The respondents did not receive any compensation for their participation.

### Data analysis

Data was analyzed using the Statistical Package for the Social Sciences (SPSS) version 22 for WINDOWS computer program (SPSS Inc. Chicago). Preliminary data analysis included descriptive statistics, i.e. means, standard deviations, frequencies and percentages for describing study population. Chi-square test for independence was done to compare the characteristics of patients attending DM clinics at secondary versus tertiary healthcare facilities. All variables with *p*-value of ≤0.05 were considered for multivariable logistic regression to determine correlates of tertiary healthcare facility. All probabilities were two-tailed and *p* values < 0.05 were regarded as significant. The dependent variable was the levels of healthcare facility namely; secondary-level health care facility and tertiary-level health care facility. Independent variables were age, type of residence, marital status, education level, duration of the disease since diagnosis, suffering from diabetes-related complications and the type of health care provided who attended them at the diabetes clinic visit.

## Results

### Demographic characteristics of the respondents by level of healthcare facility

About 180 (54.5%) of the respondents attended diabetes care clinics at a secondary-level healthcare facility. One hundred and eighty-nine (57.3%) respondents were females. The mean age of the respondents was 40.27 ± 13.31 years. Respondents from urban residence comprised 55.2% (182). Most of the respondents (66.1%) were currently married. About 57.3% (189) of the respondents were employed and 63.5% (210) had primary education. Education level differed significantly by level of healthcare facility (*p* < 0.05). Tertiary-level healthcare facility had higher proportion of respondents with secondary and tertiary education level compared to those receiving health care at the secondary-level healthcare facility (*p* = 0.000) (Table 1).

**Table 1:** Demographic characteristics of the respondents by level of healthcare facility (*N* = 330)

Demographic characteristics	Total n (%)	Level of healthcare facility		*P*-value
		Secondary n (%)	Tertiary n (%)	
Sex				0.984
Male	141 (42.7)	77 (42.8)	64 (42.7)	
Female	189 (57.3)	103 (57.2)	86 (57.3)	
Age (years) (Mean ± SD 40.27 ± 13.31)				0.055
≤ 30	95 (28.8)	42 (23.3)	53 (35.3)	
31–40	111 (33.6)	66 (36.7)	45 (30.0)	
≥ 41	124 (37.6)	72 (40.0)	52 (34.7)	
Residence				0.051
Rural	148 (44.8)	90 (50.0)	58 (38.7)	
Urban	182(55.2)	90 (50.0)	92 (61.2)	
Marital Status				0.050
Not married	112 (33.9)	70 (38.9)	42 (28.0	
Currently married	218 (66.1)	110 (61.1)	108 (72.0)	
Level of education				0.000
No formal	5 (1.5)	2 (1.1)	3 (2.0)	
Primary	210 (63.6)	135 (75.0)	75 (50.0)	
Secondary	79 (23.9)	33 (18.3)	46 (30.7)	
Tertiary	36 (10.9)	10 (5.6)	26 (17.3)	
Employment status				0.563
Not employed	141 (42.7)	80 (44.4)	61 (40.7)	
Employed	189 (57.3)	100 (55.6)	89 (59.3)	

### Clinical characteristics of the respondents by level of healthcare facility

The majority of the respondents 299 (69.4%) were diagnosed with diabetes within the recent ten years. Almost all of the respondents (93.3%) were obese. Two hundred and nine (63.3%) were hypertensive and three hundred and sixteen (95.8%) had hyperglycemia. Two-thirds of the respondents owned a glucometer. Prevalence of diabetic-related complications was 65.8%. Respondents’ duration of diabetes, presence of diabetic related complications, random blood glucose and glucometer ownership were significantly different (*p* < 0.05) by level of healthcare facility. Prevalence of recently (≤10 years) diagnosed patients was higher at the tertiary healthcare facility compared to secondary healthcare facility (83.4% versus 57.8%, *p* = 0.000). Proportion of respondents suffering from diabetes-related complications was higher among the respondents at a tertiary healthcare facility (80.7% versus 53.3% respectively, *p* = 0.000). Prevalence of hyperglycemia was slightly higher at the secondary-level healthcare facility (98.9% versus 92% respectively, *p* = 0.005). Proportion of glucometer ownership was higher at the tertiary healthcare facilities compared to the secondary facility (99.3% versus 40.6%, *p* = 0.000) (Table 2).

**Table 2:** Clinical characteristics of the respondents by level of healthcare facility (*N* = 330)

Clinical characteristics of respondents	Total n (%)	Level of healthcare facility		*P*-value
		Secondary n(%)	Tertiary n (%)	
Duration of the diabetes (years)				0.000
≤ 5	108 (32.7)	41 (22.8)	67 (44.7)	
6–10	121 (36.7)	63 (35.0)	58 (38.7)	
> 10	101 (30.6)	76 (42.2)	25 (16.7)	
Body mass index (BMI) status				1.000
Non obese	22 (6.7)	12 (6.7)	10 (6.7)	
Obese	308 (93.3)	168 (93.3)	140 (93.3)	
Blood pressure (B. P) status				
Normal	121 (36.7)	64 (35.6)	57 (38.0)	0.731
Hypertension	209 (63.3)	116 (64.4)	93 (62.0)	
Suffering from diabetes related complications^a^				0.000
No	113 (34.2)	84 (46.7)	29 (19.3)	
Yes	217 (65.8)	96 (53.3)	121 (80.7)	
Random blood glucose (RBG) status				0.005
Normal	14 (4.2)	2 (1.1)	12 (8)	
Hyperglycemia	316 (95.8)	178 (98.9)	138 (92)	
Having a glucometer				0.000
No	108 (32.7)	107 (57.4)	1 (0.7)	
Yes	222 (67.3)	73 (40.6)	149 (99.3)	

^a^*Impaired vision, lower-extremity amputations, skin ulcerations, erectile dysfunction and impaired feet sensation*

### Respondents’ perception on consultation time and health education received during routine diabetes clinic visits by level of healthcare facility

**Fig. 1:**
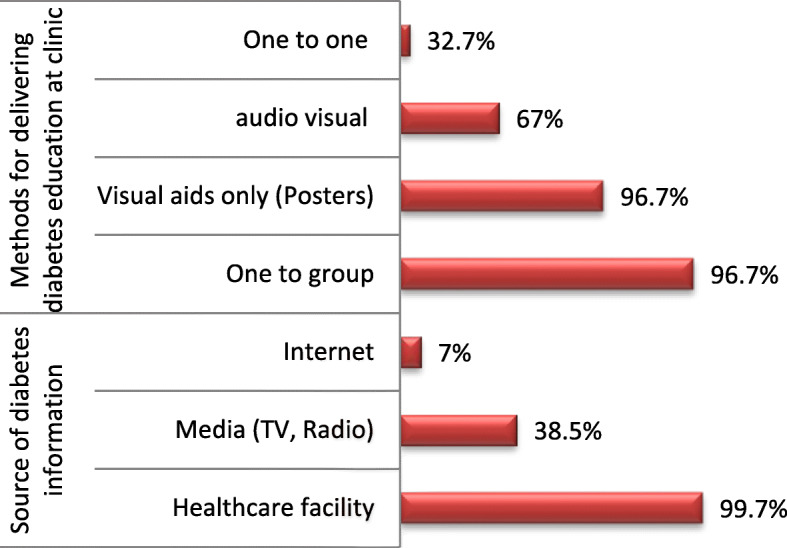
Sources of diabetes related information and mode of delivering diabetes related education

More than half (58.2%) of the respondents were not satisfied with time devoted by HCP during the consultation. Fifty-nine percent of the respondents were not satisfied with the explanations about diabetes or dietary guidance given during the clinic visits. On the other hand, most of the respondents acknowledged receiving health education when first diagnosed (96.7%) and also during routine diabetes clinic visits (97.0%). Respondents received diabetes-related information through healthcare facility (99.7%), television and radio (38.5%) and internet (7%). Diabetes-related information was received through one to group communication (96.7%), one to one (3.3%), visual aid only (67.3%) and audio-visual (32.7%) (Fig. 1).

The proportion of respondents who were satisfied with consultation time, explanation about diabetes and dietary guidance provided during the clinic visit was significantly higher at tertiary-level healthcare facility compared to the secondary healthcare facility (*p* = 0.000). Meanwhile, the proportion of respondents who acknowledged receipt of diabetes health education at diagnosis and during routine clinic visits was significant higher at the secondary healthcare facility (*p* = 0.001) (Table 3).

**Table 3:** Respondents’ perceptions of consultation time and health education received by level of healthcare facility

Perception on consultation time and health education	Total n (%)	Level of healthcare facility		*P*-value
		Secondary n (%)	Tertiary n (%)	
Satisfied with time devoted by the HCP^a^ during consultation.				
Yes	138 (41.8)	29 (16.1)	109 (72.7)	0.000
No	192 (58.2)	151 (83.9)	41 (27.3)	
Satisfied with explanation from HCP^a^ about diabetes disease.				
Yes	135 (40.9)	29 (16.1)	106 (70.7)	0.000
No	195 (59.1)	151 (83.9)	44 (29.3)	
Satisfied with explanation from HCP^a^ about dietary guidance.				
Yes	134 (40.6)	30 (16.7)	104 (69.3)	0.000
No	196 (59.4)	150 (83.3)	46 (30.7)	
Received health education when first diagnosed with diabetes.				
Yes	319 (96.7)	180 (100.0)	139 (92.7)	0.001
No	11 (3.3)	0 (0.0)	11(7.3)	
Receiving health education on routine diabetes clinic care visit.				
Yes	320 (97.0)	180 (100.0)	140 (93.3)	0.001
No	10 (3.0)	0 (0.0)	10 (6.7)	

^**a**^*Healthcare Provider*

### Health assessments received during diabetes care clinic visit

Three hundred and twenty-nine respondents (99.7%) underwent blood sugar testing, blood pressure examination and measured body weight once per month. Most respondents reported that they had their feet (98.5%) and eyes (98.2%) examined once per month. None of the respondents underwent lipid profile testing (Table 4).

**Table 4:** Respondents’ health assessments received during diabetes clinic visits (*N* = 330)

Health assessments received	Never	Weekly	Monthly
	n (%)	n (%)	n (%)
Blood sugar test	0 (0.0)	1 (0.3)	329 (99.7)
Blood pressure examination	0 (0.0)	1 (0.3)	329 (99.7)
Foot exam	5 (1.5)	0 (0.0)	325 (98.5)
Eye exam	6 (1.8)	0 (0.0)	324(98.2)
Body weight measurement	1 (0.3)	0 (0.0)	329 (99.7)
Lipid profile test	330 (100)	0 (0.0)	0(0.0)

### Type of healthcare provider during diabetes clinic visits by level of healthcare facility

The respondents received their care from nurses (100%), a clinician (49.7%) and a nutritionist/dietitian (23%) during routine diabetes clinic visits. Regarding the levels of healthcare facility, the proportion of respondents attended by a clinician was significantly higher at secondary compared to tertiary-level healthcare facility (59.4% versus 38.0%, *p* = 0.000). In all settings the clinician was either a medical doctor, clinical officer or assistant medical officer. On the other hand, proportion of respondents attended by nutritionist or dietitian was significantly higher at tertiary-level healthcare facility compared to secondary healthcare facilities (29.3% versus 17.8%, *p* = 0.019) (Table 5).

**Table 5:** Healthcare provider during diabetes clinic visits by level of healthcare facility (*N* = 330)

Healthcare provider	Total	Level of healthcare facility		*P* - value
	n (%)	Secondary – n (%)	Tertiary – n (%)	
Nurse				
Yes	330 (100)	180 (100)	150 (100)	*
No	0 (0)	0 (0)	0 (0)	
Clinician**				
Yes	164 (49.7)	107 (59.4)	57 (38.0)	0.000
No	166 (50.3)	73 (40.6)	93 (62.0)	
Nutritionist/dietitian				
Yes	76(23.0)	32 (17.8)	44 (29.3)	0.019
No	254 (77.0)	148 (82.2)	106 (70.7)	

* not calculated, ** medical doctor, clinical officer and assistant medical officer

### Correlates of tertiary-level healthcare facility among respondents attending diabetes clinic care visit

A total of eight variables from Tables 1, 2 and 5 with *p*-value ≤0.05 and appropriate for multivariable analysis were further analyzed in the multivariable logistic regression model (Table 6). After adjusting, the respondent’s age, marital status, duration of diabetes, suffering from diabetes related diseases and being attended by a nutritionist were significant and associated with tertiary-level healthcare facility. The odds of attending clinic at a tertiary-level healthcare facility decreased with age. Compared to respondents aged ≥41 years, the odds of attending clinic at tertiary-level healthcare facility were AOR = 6.67; 95% CI: 2.86, 17.39 for respondents aged ≤30 years and AOR = 1.93; 95% CI: 1.03, 3.87 for respondents aged 31–40 years. Married respondents were 2.8 times more likely to attend clinic at tertiary healthcare facility rather than the secondary healthcare facility (AOR = 2.83; 95% CI: 1.49, 5.37). Compared with the respondents with diabetes for ≥11 years since diagnosis, the odds of attending clinic at a tertiary-level healthcare facility were AOR = 11.11; 95% CI: 4.77, 25.37 for respondents with diabetes for ≤5 years and AOR = 2.13; 95% CI: 1.03,4.38 for respondents with diabetes for 6–10 years. Respondents suffering from diabetes related complications were 4.95 times more likely to attend clinic at a tertiary-level healthcare facility. Regarding the HCP, respondents at tertiary healthcare facility were three times and more likely to receive health care from a nutritionist/ dietitian and less likely to receive health care from a clinician than those receiving their care at secondary health care facilities.

**Table 6:** Correlates of tertiary-level healthcare facility among respondents attending diabetes clinic care visit

Respondents’ characteristics	AOR	95% C. I for AOR		*P*-value
		Lower	Upper	
Age (years)				
≤ 30	6.67	2.87	17.39	.000
31–40	1.93	1.03	3.87	.001
≥ 41	1			
Type of residence				
Rural	1			
Urban	1.48	.82	2.69	.196
Marital status				
Not Married	1			
Married	2.83	1.49	5.37	.001
Education level				
Informal and Primary	1			
Secondary	1.00	0.47	2.12	.996
Tertiary	3.73	1.84	7.59	.000
Duration of diabetes (years)				
≤ 5	11.11	4.77	25.37	.000
6 – 10	2.13	1.03	4.38	.041
≥ 11	1			
Suffering from diabetes related complications				
No	1			
Yes	4.95	2.51	9.76	.000
Received health care from Nutritionist/dietitian				
No	1			
Yes	3.16	1.39	7.15	.006
Received health care from a Clinician				
No	7.14	3.47	14.26	.000
Yes	1			

## Discussion

This study was designed to assess the clinical characteristics and health care received among patients with type 2 diabetes attending secondary and tertiary healthcare facilities. The current study demonstrated that patients from urban residence were more likely to attend clinic at tertiary level healthcare facility. The possible explanation for this finding could be the access to the healthcare facility. In Tanzania, diabetes patients are free to attend the clinic at any healthcare facility based on convenience. This approach seeks to reduce barriers such as long distances to the hospital, financial constrains [8] and long queues at the clinics [10] which might interfere their management plan. In our settings, tertiary healthcare facilities are mostly found in urban areas [10].

In this study, most of respondents (69.4%) were diagnosed with diabetes within the recent 10 years. Similar findings have been reported from other studies conducted in Tanzania and Pakistan [12, 14, 24]. One possible explanation for the relatively short duration of disease could the death of patients with longer disease duration. Previous studies in SSA have demonstrated that longer duration of diabetes increases the risk of developing diabetes-related complications [1, 25] that are responsible for high rates of mortality of diabetes patients in the region [1, 15, 26]. Another possible reason for this finding could be that patients with longer duration diabetes may not attend regular clinic visits for various reasons including; costs associated with visits and lack of symptoms. A previous study has demonstrated that patients with diabetes have a tendency to avoid diabetes-related medical care costs such as transportation to the facility, consultation n fees and laboratory costs [8]. These individuals may purchase medicines directly from the pharmacies, skip clinic visits and adjust their medications independently [8]. These habits are more likely to be found among patients with longer duration of diabetes compared to newly diagnosed patients.

The current study also noted that, the tertiary-level healthcare facilities had higher proportions of respondents who were recently (≤10 years) diagnosed with diabetes compared to secondary healthcare facilities. A possible explanation for this finding could be that those patients had complications when first diagnosed or developed complications shortly after diagnosis [10, 26] and were referred to tertiary healthcare facility for advanced treatment and management of those complications [7, 8]. Another possible explanation for this finding could be the fact that the availability of diabetes medication and laboratory reagents in lower healthcare facilities is often unreliable [7, 8]. When this happens, all diabetes patients may be referred to tertiary-level healthcare facilities for diagnosis and treatment [7]. Furthermore, the current finding also shows that relatively young patients were more likely to attend clinic at tertiary-level health care facility. The possible explanation for this finding could be that most of those patients were diagnosed recently and had complications [10, 26].

Controlling hyperglycemia is important for prevention of disease progression and diabetes-related complications [1]. In this study majority of the patients had hyperglycemia. Similar findings have been reported in Dar es Salaam, Tanzania [12, 22] and China [27]. Factors associated with poor glycemic control among patients with diabetes are complex involving both patients and healthcare provider related factors. The high prevalence obesity observed in this study could possibly contribute to this finding. A study conducted in Dar es Salaam that obesity was associated with poor glycemic control among patients with diabetes [12]. Increased fat mass and visceral adiposity is a known factor of insulin resistance. Another explanation could be poor medication adherence which is a major challenge facing diabetes patients in Tanzania [8, 12, 22]. Furthermore, the current study also found that hyperglycemia was slightly higher at secondary compared to tertiary-level healthcare facility. This could be due to insufficient supply of diabetes medications at lower-levels of healthcare facilities in most of our settings [7, 8]. Another possible explanation for this finding may be due to lack of specified diabetes team who are designed to deliver health care services at diabetes clinic in secondary healthcare facilities. In most secondary healthcare facilities diabetes services are provided by the same clinician who also provides care at the general outpatient department [7]. This has been reported to contribute to longer waiting hours at the clinic as the clinician has to complete morning rounds first before going to the diabetes clinic thus causing inconvenience and contributing to interruptions in medication use [8] which may lead to hyperglycemia.

This study found that prevalence of diabetes related complications was generally high. This could be a result of uncontrolled blood glucose observed in this study. It is widely known that complications in diabetes occur as a result of the injurious effects of hyperglycemia [1]. Another reason could be delayed diagnosis of diabetes as a result many patients often have complications at the time of diagnosis [26]. Our finding is in agreement with previous studies in Tanzania and globally [12–14, 28]. For instance, prior studies conducted in Tanzania have demonstrated that more than 50 % of patients with diabetes had complications [12, 13]. However, previous studies looked generally at the presence of these complications regardless the levels of the healthcare facility. As anticipated, the prevalence of diabetes related complications was much higher among the patients attending diabetes clinic at tertiary-level healthcare facility. This finding can be explained by the fact that diabetes patients with complications are usually referred to tertiary-level healthcare facility for proper treatment and management of their conditions [7, 23].

The glucometer is an essential technology for self-monitoring of blood glucose. It is a recommended and acceptable technology to facilitate the attainment of glycemic control among patients with diabetes. Similar to a study that was conducted in Karachi, Pakistan which found that 69 % of the patients attending diabetes clinic care had glucometers [29], this study found 67% of patients owned a glucometer. As expected, patients at the tertiary healthcare facility had a higher proportion of respondents who owned glucometers. Previous studies have clearly shown that, tertiary healthcare facilities are more equipped with diagnostic tools and supplies for diabetes management compared to the secondary healthcare facilities [7, 9]. Another possible reason could be that the tertiary healthcare facilitiesare found in urban areas with increased availability medical shops [10] where patients can purchase glucometers. In contrast, secondary healthcare facilities are mostly found in remote rural areas where such medical shops are rarely available. Furthermore, the types of services that are provided in these healthcare facilities could also be among the possible explanation for this finding. A glucometer is especially important for patients using insulin injections to manage their diabetes. Insulin is available at tertiary-level healthcare facilities and limited at secondary healthcare facilities in Tanzania [7, 10]. Contrary to our finding, a study from urban Pakistan found a low proportion (23%) of patients owning glucometer at home [24]. This difference may be due to the differences in study settings. The study in Pakistan was conducted at the community level and therefore likely to involve mixture of patients who may not be adhering properly to the treatment regime unlike the current study which was conducted in a clinic setting where patients are likely to be those who are adhering properly to treatment regime including having a glucometer.

In this study, the majority of patients were not satisfied with the amount of time spent by the HCP during the consultation. This finding could be explained by the heavy workload of the HCPs in most of our settings [7, 8, 10]. In addition, patients were also not satisfied with the explanation for diabetes and dietary guidance given during DM clinic visits. Lack of specific diabetes training among the HCPs and lack of guidelines and manuals for management and treatment of diabetes [7, 9] in most of our healthcare facilities could be a possible explanation of this finding. Patient satisfaction about consultation time and dietary guidance given during the clinic visit was higher among respondents attending DM clinic at the tertiary healthcare facilities. Patient counseling and health education require adequate health infrastructure and planning. Tertiary healthcare facilities are typically more equipped with specific educational materials and guidelines for the management of diabetes compared to secondary healthcare facilities [7, 9]. Additionally, the presence of a specialized team for diabetes health care services at tertiary healthcare facilities may facilitate provision of quality services compared to secondary healthcare facilities where there is no such team. In those settings, the same HCPs are responsible for providing diabetes care services at the diabetes clinic and also serving many other patients with a range of health care concerns [7, 8]. This could result in inadequate time dedicated to patient care.

Tanzania’s diabetes management guidelines specify that patients receive health education for self-care during diagnosis and at every clinic visit [23] and in this study we found that, most of the patients (> 96%) received health education. This is contrary to a study done in Dar es Salaam, Tanzania whereby only 48% of the patients received health education [11]. However, the Dar es Salaam study assessed specific health education on foot care while the current study examined generally on diabetes health education. Interestingly, the current study found that all respondents (100%) at the secondary healthcare facility received health education as per national guidelines. This may be because the secondary-level healthcare facility is where the patients usually receive initial diabetes diagnosis and management. In this setting, health education is prioritized as part of the management of diabetes package for initial management and prevention of complications. In contrast, the care focus of tertiary-level healthcare facilities is the management of diabetic patients with complications [7, 8]. It is expected that the complexity and acuity of patients with diabetes at tertiary healthcare facilities may be increased compared to those who receive care at lower level facilities [7] which hinders provision of health education.

The current study also found that most patients received diabetes-related information from the healthcare facility. Thus, patients who do not attend the clinic may not have an opportunity to receive health education. Therefore, based on normal clinic schedules, it gives an opportunity for the patient to receive health education once per month only. According to the World Health Organization (2016), mobile phones can also help in diabetes management [1]. Information through novel mobile technologies can be timely, frequent and reach many people. In Tanzania at least 64% of households have an access to mobile phone [30]. As such, it is imperative that we explore effective use of mobile technology in management of diabetes as well as other diseases.

Regular in-person clinic visits are crucial for providing optimal care of patients with diabetes [1]. In this study that was conducted within health care facilities, we found that the majority of respondents (> 98%) attended regular clinic visits where they underwent monthly assessment of blood sugar, blood pressure, examination of eyes and feet and weight assessment. This matches the national guidelines for management of diabetes which advises this scope of care at diagnosis and on a monthly basis [23]. However, none of the patients in our study had ever undergone blood lipid testing despite national recommendation for such testing at initial diagnosis and then at least annually for patients with normal values [23]. The unavailability of equipment and supplies for lipid analysis [7] and lack of trained staff to run such machines [19] in most healthcare facilities in our setting may explain this finding. Lack of screening and treatment for hyperlipidemia likely increases cardiovascular disease risk as diabetes is a significant risk factor for dyslipidemia and heart disease [1]. The current finding is in agreement with the finding of Kamuhabwa and Charles who found that less than 2% of patients with diabetes had a record of lipid profile testing [12].

This study indicates that, all respondents received health care with a nurse during diabetes care clinic visits. Similarly, the study conducted in Dar es Salaam showed that 83.5% of diabetes patients were receiving diabetes medical care services from nurses [11]. This finding indicates that nurses are the key players in provision of diabetes health care services at diabetes clinics in our healthcare facilities. The higher number of nurses relative to other healthcare professionals in our healthcare facilities [7, 9] could be a reason for this finding. Additionally, we also observed that the proportion patients with diabetes who received health care with a clinician were significantly higher at secondary-level healthcare facilities. This finding is in agreement with the study done by Mwangombe et al., who reported that, clinicians are the main HCPs of diabetes health care services at lower healthcare facilities while at tertiary-level healthcare facilities diabetes services are being provided by a team of professionals which is comprises of clinicians, nurses and a coordinator [7]. We also observed that very few patients had the opportunity to receive care with a nutritionist or dietitian during their diabetes clinic visit. This is contrary to the World Health Organization recommendations which advise a range of health professionals for the care and treatment of diabetes, including: physicians, nurses, dietitians and other medical specialists [1]. Unavailability of a nutritionist or dietitian for diabetes health care can be explained by the shortage of the cadre in our healthcare settings [7]. In addition the few nutritionists available at the healthcare facility do not feel confident to provide such care [7]. In-service training and staff recruitment are recommended at the healthcare system to enable delivery of high quality diabetes care services in our settings.

## Conclusions

Overall, the current study observed a high prevalence of hyperglycemia, obesity, diabetes related complications and hypertension among T2DM patients attending diabetes clinics at healthcare facilities in Mwanza Region. Optimal lifestyle practices should continue to be emphasized during patient education. Intervention on obesity should be given priority among the patients with diabetes. Assessment of blood pressure, blood glucose, weight, feet and eye were performed on monthly basis as per national guidelines. However, none of the respondents had undergone lipid profile test. Nurses were the most common HCPs at diabetes clinics. Prevalence of glucometer ownership, diabetes related complications, satisfaction of diabetes services received at the clinic, random blood glucose and duration of diabetes was significant different by levels of healthcare facility. Healthcare systems should be strengthened to ensure that every diabetes clinic is well equipped with diabetes health care team, equipment and supplies to provide adequate health care services.

### Strengths and limitations

This is the first study in Tanzania to explore the characteristics of patients with T2DM attending clinics at different levels of healthcare facility. This information will provide insight into a variety of strategies for improving the diagnosis and management of patients with T2DM in these settings. This study had a number of limitations. The study did not assess at the quality of care that patients with diabetes receives at the clinic. We did not assess the level or content of diabetes knowledge among the patients. We used random blood glucose to assess hyperglycemia and the study was conducted in hospital settings.

## Supplementary information

**Additional file 1.** Questionnaire: Assessment of clinical characteristics and health care services received among type 2 diabetes mellitus patients attending specialized diabetes clinics at secondary and tertiary health care facilities in Mwanza Region, Tanzania: A cross-sectional study. The questionnaire shows questions used to collect information on clinical characteristics and health care services received among type 2 diabetes mellitus patients attending specialized diabetes clinics.

## Data Availability

The datasets used and/or analysed during the current study are available from the corresponding author on reasonable request.

## Abbreviations

BMC: Bugando Medical Centre
DM: Diabetes Mellitus
HCP: Healthcare Provider
T2DM: Type 2 Diabetes Mellitus
SSA: Sub-Saharan African
WHO: World Health Organization

## References

[1] World Health Organization. Global Report on Diabetes. WHO 2016 https://www.who.int/diabetes/global-report/en/ Accessed on 8th March, 2019.

[2] IDF: International Diabetes Federation. DIABETES ATLAS 6th edition. 2013 http://www.idf.org.diabetesatlas Accessed on 5th March, 2019.

[3] IDF: International Diabetes Federation. DIABETES ATLAS 7th edition. 2015 https://www.idf.org/our-activities/advocacy-awareness/resources-and-tools/13:diabetes-atlas-seventh-edition.html Accessed on 5th March, 2019.

[4] Chiwanga FS, Njelekela MA, Diamond MB, Bajunirwe F, Guwatudde D, Nankya-Mutyoba J, Kalyesubula R, Adebamowo C, Ajayi IO, Reid TG, Volmink J, Laurence C, Adami H, Holmes MD, Dalal S (2016). Urban and rural prevalence of diabetes and pre-diabetes and risk factors associated with diabetes in Tanzania and Uganda. Glob Health Action.

[5] Lunyera J, Wang D, Maro V, Karia F, Boyd D, Omolo J, Patel UD (2016). Stanifer JW and for the comprehensive kidney disease assessment for risk factors, epidemiology, knowledge, and attitudes (CKD AFRiKA) study: traditional medicine practices among community members with diabetes mellitus in northern Tanzania: an ethnomedical survey. BMC Complement Altern Med.

[6] Ruhembe CC, Mosha TC, Nyaruhucha CN (2014). Prevalence and awareness of type 2 diabetes mellitus among adult population in Mwanza city, Tanzania. Tanzan J Health Res.

[7] Mwangome M, Geubbels E, Klatser P, Dieleman M (2016). Perceptions on diabetes care provision among health providers in rural Tanzania: a qualitative study. Health Policy Plan.

[8] Metta E, Haisma H, Kessy F, Geubbels E, Hutter I, Bailey A (2015). “It is the medicines that keep us alive”: lived experiences of diabetes medication use and continuity among adults in Southeastern Tanzania. BMC Health Serv Res.

[9] Peck R, Mghamba J, Vanobberghen F, Kavishe B, Rugarabamu V, Smeeth L, Hayes R, Grosskurth H, Kapiga S (2014). Preparedness of Tanzanian health facilities for outpatient primary care of hypertension and diabetes: a cross-sectional survey. Lancet Glob Health.

[10] Kolling M, Winkley K, Deden MV (2010). “For someone who’s rich, it’s not a problem”. Insights from Tanzania on diabetes health-seeking and medical pluralism among Dar es Salaam’s urban poor. Globalization Health.

[11] Chiwanga FS, Njelekela MA (2015). Diabetic foot: prevalence, knowledge, and foot self-care practices among diabetic patients in Dar Es Salaam, Tanzania–a cross-sectional study. J Foot Ankle Res.

[12] Kamuhabwa AR, Charles E (2014). Predictors of poor glycemic control in type 2 diabetic patients attending public hospitals in Dar es Salaam. Drug Healthcare Patient Safety.

[13] Stanifer JW, Cleland CR, Makuka GJ, Egger JR, Maro V, Maro H, Karia F, Patel UD, Burton MJ, Philippin H (2016). Prevalence, risk factors, and complications of diabetes in the Kilimanjaro region: a population-based study from Tanzania. PLoS One.

[14] Damian DJ, Kimaro K, Mselle G, Kaaya R, Lyaruu I (2017). Prevalence of overweight and obesity among type 2 diabetic patients attending diabetes clinics in northern Tanzania. BMC research notes.

[15] Hall V, Thomsen RW, Henriksen O, Lohse N (2011). Diabetes in sub Saharan Africa 1999-2011: epidemiology and public health implications. A systematic review. BMC Public Health.

[16] Shrivastava S, Shrivastava PS, Ramasamy J (2013). Role of self-care in management of diabetes mellitus. J Diabetes MetabDisord.

[17] Kavishe B, Biraro S, Baisley K, Vanobberghen F, Kapiga S, Munderi P, Smeeth L, Peck R, Mghamba J, Mutungi G, Ikoona E, Levin J, Monclús MAB, Katende D, Kisanga E, Hayes R, Grosskurth H (2015). High prevalence of hypertension and of risk factors for non-communicable diseases (NCDs): a population based cross-sectional survey of NCDS and HIV infection in northwestern Tanzania and southern Uganda. BMC Med.

[18] Muhihi A, Njelekela M, Mpembeni R, Masesa Z, Kitamori K, Mori M, Kato N, Mtabaji J, Yamori Y (2012). Physical activity and cardiovascular disease risk factors among young and middle-aged men in urban Mwanza, Tanzania. Pan Afr Med J.

[19] Njelekela MA, Mpembeni R, Muhihi A, Mligiliche NL, Spiegelman D, Hertzmark E, Liu E, Finkelstein JL, Fawzi WW, Willet WC, Mtabaji J (2009). Gender-related differences in the prevalence of cardiovascular disease risk factors and their correlates in urban Tanzania. BMC Cardiovasc Dis.

[20] United Republic of Tanzania: National Bureau of Statistics (2013). The 2012 Population and housing census population distribution by administrative areas. Dar es Salaam.

[21] United Republic of Tanzania (2011). The Tanzania Economic Survey 2010.

[22] Rwegerera G (2014). M: adherence to anti-diabetic drugs among patients with type 2 diabetes mellitus at Muhimbili National Hospital, Dar es salaam, Tanzania- A cross-sectional study. Pan Afr Med J.

[23] United Republic of Tanzania: Ministry of health and social welfare (2017). Standard treatment guidelines and essential medicines list. Fifth edition.

[24] Ahmed MU, Seriwala HM, Danish SH, Khan AM, Hussain M, Husain M, Ahmed MM, Anis K (2016). Knowledge, attitude, and self care practices Amongsts patients with type 2 diabetes in Pakistan. Global J Health Sci.

[25] Nelson RG, Bennett PH, Beck GJ, Tan M, Knowler WC, Mitch WE, Hirschman GH, Myers BD (1996). Development and progression of renal disease in Pima Indians with non-insulin-dependent diabetes mellitus. N Engl J Med.

[26] Kengne AP, Amoah AG, Mbanya JC (2005). Cardiovascular complications of diabetes mellitus in sub-Saharan Africa. Circulation..

[27] Xu Y, Wang L, He J, Bi Y, Li M, Wang T, Wang L, Jiang Y, Dai M, Lu J, Xu M, Li Y, Hu N, Li J, Mi S, Chen CS, Li G, Mu Y, Zhao J, Kong L, Chen J, Lai S, Wang W, Zhao W, Ning G (2013). 2010 China Noncommunicable Disease Surveillance Group**.**Prevalence and control of diabetes in Chinese adults. JAMA.

[28] Kohner EM, Aldington SJ, Stratton IM, Manley SE, Holman RR, Matthews DR, Turner RC (1998). United Kingdom prospective diabetes study, 30: diabetic retinopathy at diagnosis of non–insulin-dependent diabetes mellitus and associated risk factors. Arch Ophthalmol.

[29] Badruddin N, Basit A, Hydrie MZ, Hakeem R (2002). Knowledge, attitude and practices of patients visiting a diabetes care unit. Pak J Nutr.

[30] National Bureau of Statistics (NBS) and Office of Chief Government Statistician (OCGS), Zanzibar. 2014. The 2012 population and housing census: basic demographic and socio- economic profile; key findings. Dar es Salaam: NBS and OCGS.

