# Supplementary material for: Clinical characteristics and health care received among patients with type 2 diabetes attending secondary and tertiary healthcare facilities in Mwanza Region, Tanzania: a cross-sectional study
Source: BMC Health Serv Res. 2020 Jun 10;20:527. doi: 10.1186/s12913-020-05407-y (PMC7288541; doi:10.1186/s12913-020-05407-y)
Supplement: Supplementary file 1 — Additional file 1. Questionnaire: Assessment of clinical characteristics and health care services received among type 2 diabetes mellitus patients attending specialized diabetes clinics at secondary and tertiary health care facilities in Mwanza Region, Tanzania: A cross-sectional study. The questionnaire shows questions used to collect information on clinical characteristics and health care services received among type 2 diabetes mellitus patients attending specialized diabetes clinics. [file 12913_2020_5407_MOESM1_ESM.docx]

# Appendix 1: Questionnaire on assessment of clinical characteristics and health care services received among type 2 diabetes mellitus patients attending specialized diabetes clinics at secondary and tertiary health care facilities in Mwanza Region, Tanzania: A cross-sectional study”.

**INSTRUCTIONS**

1. This questionnaire consists of **four (4) Parts**
2. For all questions, fill the most correct answer (s) in the spaces provided

**Introduction**

Date of interview: ………… /……………/2018

Name of researcher/interviewer.............................

Questionnaire number……………………………

District...................................................................

Ward ………………………………....................

Name of healthcare facility………………………………

**PART One:** Demographic characteristics of the respondents (Questions 1 – 6)

1. Age of the respondents (years) ________________
2. Sex
3. Male ( )
4. Female ( )
5. Type of home residence
6. Rural
7. Urban
8. Marital status
9. Currently married ( )
10. Single ( )
11. Divorced ( )
12. Widow ( )
13. Level of education
14. Informal ( )
15. Primary ( )
16. Secondary ( )
17. College/university ( )
18. Occupation
19. Formal Employment ( )
20. Self-employment ( )
21. Farmer/Peasant ( )
22. Household chores ( )

**PART Two:** Clinical information of the patient (Questions 7 – 18)

1. Type of healthcare facility
   1. Secondary
   2. Tertiary
2. When were you diagnosed with diabetes (Year) _________
3. What was your age when you were diagnosed with diabetes (Age)__________
4. Do you have your own glucometer ?
   1. Yes
   2. No
5. Do you have any complications related to diabetes?
6. Yes ( ) Mention ………………………………………………………………...

…………………………………………………………………………………………..

1. No ( )
2. Do you smoke cigarette?
3. Yes ( )
4. No ( )
5. Do you take alcohol?
6. Yes ( )
7. No ( )
8. Measured body height (cm) ___________
9. Measured body weight (kg) ______________
10. Measured diastolic blood pressure (mmHg)_______________
11. Measured Systolic blood pressure (mmHg)________________
12. Measure blood sugar level (mmol/l)_________________

**PART Three:** Perceptions on diabetes health care services received during clinic visit (Questions 19 – 26)

1. Do you get satisfied with the time devoted for consultation by the health care provider during clinic visit?
2. Yes ( )
3. No ( )
4. Do you get satisfied with the explanation about diabetes mellitus disease given to you by the health care provider during clinic visit?
5. Yes ( )
6. No ( )
7. Do you get satisfied with the explanation about the diet recommended for patients with diabetes mellitus disease given to you by the health care provider during clinic visit?
8. Yes ( )
9. No ( )
10. When diagnosed with diabetes mellitus for the first time, were you given any health education about diabetic mellitus?
11. Yes ( )
12. No ( )
13. When attending regular diabetes mellitus clinic, are you provided with any kind of health education about diabetes mellitus?
14. Yes ( )
15. No ( )
16. How were you given the health education at the health care facility (multiple response)
17. One to one communication
18. One to group communication
19. By using audio-visual eg TV
20. By using audio means only e.g Radio
21. By using visual eg Posters, Diagram etc
22. Others
23. During routine diabetes clinic visit, who normally attends you? (Multiple response)?
24. Nurse ( )
25. Dietitian/ Nutritionist ( )
26. Medical doctor ( )
27. Others ( ) Mention ……………………………
28. Where do you get information about diabetes mellitus (Multiple response)?
29. Health care facility ( )
30. Media ( ) Mention ………………………
31. Internet ( )
32. Other, specify ……………………………………………………….

**PART Four:** Health assessments performed during diabetes care clinic visit (question 27 – 32)

1. For the past three months, how often did you perform blood sugar examination at the health care facility
2. Never ( )
3. Weekly ( )
4. Monthly ( )
5. Others ( ) Specify ………………………
6. For the past three months, how often did you measure your blood pressure at the heath care facility?
7. Never ( )
8. Weekly ( )
9. Monthly ( )
10. Others ( ) Specify ………………………
11. For the past three months, how often did you measure your lipid profile at the heath care facility?
12. Never ( )
13. Weekly ( )
14. Monthly ( )
15. Others ( ) Specify ………………………
16. For the past three months, how often have you had your feet examined at the heath care facility?
17. Never ( )
18. Weekly ( )
19. Monthly ( )
20. Others ( ) Specify ………………………
21. For the past three months, how often have you had your eyes examined at the heath care facility?
22. Never ( )
23. Weekly ( )
24. Monthly ( )
25. Others ( ) Specify ………………………
26. For the past three months, how often have you had your weight measured at the heath care facility?
27. Never ( )
28. Weekly ( )
29. Monthly ( )
30. Others ( ) Specify ………………………

**Stay blessed**
